# Green tea (*Camellia sinensis*) aqueous extract alleviates postmenopausal osteoporosis in ovariectomized rats and prevents RANKL-induced osteoclastogenesis *in vitro*

**DOI:** 10.29219/fnr.v62.1478

**Published:** 2018-10-08

**Authors:** Xin Wu, Chuan-qi Xie, Qiang-qiang Zhu, Ming-yue Wang, Bin Sun, Yan-ping Huang, Chang Shen, Meng-fei An, Yun-li Zhao, Xuan-jun Wang, Jun Sheng

**Affiliations:** 1 Key Laboratory of Pu-erh Tea Science, Ministry of Education, Yunnan Agricultural University, Kunming, China; 2 College of Food Science and Technology, Yunnan Agricultural University, Kunming, China; 3 State Key Laboratory of Phytochemistry and Plant Resources in West China, Kunming Institute of Botany, Chinese Academy of Sciences, Kunming, China; 4 College of Science, Yunnan Agricultural University, Kunming, China; 5 State Key Laboratory for Conservation and Utilization of Bio-Resources in Yunnan, Kunming, China

**Keywords:** green tea aqueous extract, osteoporosis, ovariectomy, receptor activator of the nuclear factor kappa B ligand, osteoclast

## Abstract

**Background:**

Green tea (*Camelliasinensis* [L.] Kuntze) belongs to the plant family Theaceae and is mainly distributed in East Asia, the Indian subcontinent and Southeast Asia. This plant has been proven to be beneficial to human health, and green tea is the second most consumed beverage in the world after water. However, until now, the effect of green tea aqueous extract (GTE) upon postmenopausal osteoporosis has remained unclear. In this study, we investigated the therapeutic effects of GTE on estrogen deficiency-induced osteoporosis and explored the possible mechanisms *in vivo* and *in vitro*.

**Materials and methods:**

Ovariectomized (OVX) female rats were orally administered with GTE at doses of 60, 120, and 370 mg kg^−1^ for 13 consecutive weeks. The biochemical parameters, bone gla protein, alkaline phosphatase, acid phosphatase, estrogen, interleukin-1β, and interleukin-6 in blood samples were detected, and histological change in bones was analyzed by hematoxylin and eosin staining. Meanwhile, the mechanisms of GTE on osteoclast formation were explored in RAW 264.7 cells induced by receptor activation of the nuclear factor kappa B ligand (RANKL).

**Results:**

The results showed that GTE could increase bone mass and inhibit trabecular bone loss in OVX rats. Furthermore, real-time quantitative reverse transcription polymerase chain reaction analysis from *in vitro* experiments also showed that GTE reduced the mRNA expression of osteoclast-associated genes such as *cathepsin K* (cath-K), *c-Fos*, *matrix metalloproteinase 9*, *nuclear factor of activated T cells cytoplasmic 1* (NFATc1) and *tartrate-resistant acid phosphatase*. In addition, GTE caused a reduction in the protein levels of NFATc1, c-Fos, c-src and cath-K.

**Conclusion:**

Evidence from both animal models and *in vitro* experiments suggested that GTE might effectively ameliorate the symptoms of osteoporosis in OVX rats and inhibit RANKL-induced osteoclast-specific gene and protein expression.

Osteoporosis is a systemic metabolic bone disease characterized by low bone mass, damaged microstructure, highly fragile bone, and greater vulnerability to fracture (1). The factors underlying osteoporosis are very complex and involve aging, endocrine disorders, calcium malabsorption, and limb disuse, as well as immune, nutritional, and genetic factors (2). There are two types of osteoporosis: primary and secondary. Postmenopausal osteoporosis (PMOP) is the most prevalent of the primary forms of osteoporosis (3). Bone metabolism is a process of dynamic equilibrium, in which osteoblasts and osteoclasts work together to maintain homeostasis. However, the dynamic equilibrium of bone metabolism can be disrupted in response to the reduced level of estrogen (4).

Postmenopausal women are at high risk of developing osteoporosis because of the significant alterations in bone metabolism associated with estrogen deficiency (5). Approximately half of women over the age of 50 years are expected to suffer an osteoporosis-related fracture over their remaining lifetime (6, 7). Some individuals may develop osteopenia, a condition characterized by low bone density (8). The rapid bone loss and higher bone fragility that take place during menopause lead to an increased incidence of spine, hip, and wrist fractures in postmenopausal women. Estrogen replacement therapy (9), which represents the most common therapy for the prevention and treatment of PMOP, has been reported to be associated with an increased risk of breast cancer, ovarian cancer, endometrial cancer, and cardiovascular disease in postmenopausal women (10). Therefore, estrogen therapy is no longer recommended for the prevention of fractures in postmenopausal women, and developing alternative treatment strategies is needed (11).

Thousands of years of human experimentation has led to a significant belief in the safety of ‘natural’ products and this has contributed to the fairly widespread use of complementary therapies to relieve postmenopausal symptoms (12). Since ancient times, green tea, a Chinese traditional beverage, has been held to be beneficial to human health. The pharmacological effects and safety of this plant have been confirmed, particularly in respect of catechins, which make up 30% of the dry weight of tea leaves (13). Water extracts from green tea contain abundant bioactive constituents and have shown various biological activities, including antioxidant (14), anti-obesity (15), hypolipidemic, antidiabetic (16), anti-inflammatory, and anticancer (17) properties. Tea drinking is closely associated with bone health and may provide protection against osteoporosis and osteoporotic fracture; these effects have been verified both *in vitro* and *in vivo* (13, 18–20). Previous studies also showed that some chemical compositions of tea could improve bone loss *in vivo* (15, 21). Green tea polyphenols could improve bone loss in middle-aged female rats (21). (-)-Epigallocatechin-3-gallate, a main active ingredient in green tea, also showed a protective effect on bone microarchitecture in ovariectomized (OVX) rats (15). However, until now, the effect of green tea aqueous extract (GTE) upon PMOP has not been specifically investigated.

In the present study, we demonstrate that GTE had an ameliorative effect in OVX-induced osteoporosis rats, and that GTE could inhibit osteoclastic activities *in vitro*. Collectively, these data provide a theoretical foundation relating to the molecular mechanisms of GTE against osteoporosis.

## Materials and methods

### Reagents and antibodies

*Escherichia coli (E. coli)*-derived recombinant mouse receptor activation of the nuclear factor kappa B ligand (RANKL) was purchased from R&D Systems (Minneapolis, MN, USA) and dissolved in 1% bovine serum albumin in phosphate-buffered saline. Xian-Ling-Gu-Bao (XLGB) capsules were obtained from Guizhou Tongjitang Pharmaceutical Co., Ltd. (Guizhou, China). Alkaline phosphatase (ALP), calcium (Ca), and phosphorus (P) assay kits were purchased from Zhong-Sheng BeiKong Bio-Technology and Science (Beijing, China). Bone gla protein (BGP) and estrogen (E_2_) radioimmunoassay kits were obtained from the Beijing North Institute of Biological Technology (Beijing, China). Rat interleukin-1β (IL-1β) and IL-6 enzyme-linked immunosorbent assay kits (R111102-07a; R111102-06a) were purchased from NeoBioscience Biological Technology Co., Ltd. (Shenzhen, China). tartrate-resistant acid phosphatase (TRAP) staining kits and acid phosphatase (ACP) assay kits were obtained from Nanjing Jiancheng Bioengineering Institute (Nanjing, China). Anti-nuclear factor of activated T cells cytoplasmic 1 (anti-NFATc1), anti-c-Src, anti-cathepsin K, and anti-c-Fos antibodies were purchased from Santa Cruz Biotechnology (Santa Cruz, CA, USA). Anti-β-tubulin and horseradish peroxidase-conjugated secondary antibodies were purchased from Proteintech Group, Inc. (Rosemont, IL, USA) and Thermo Fisher Scientific (Waltham, MA, USA), respectively.

### Preparation of GTE

The green tea from a variety named Yunnan Daye (*Camellia sinensis* [Linn.] var. assamica [Masters] Kitamura) (22) were collected in 2015 in Yunnan Province and identified by Prof. Kaicong Fu of the Pu’er National Institute of Traditional Medicine, and a voucher specimen (2015-DPE-5) was deposited in the Key Laboratory of Pu-erh Tea Science of Yunnan Agricultural University. In each case, 100 g of green tea was extracted twice using 1200 mL of water each time (1.5 hours) under reflux. The extract was then decanted, filtered, and vacuum freeze-dried to obtain a 20 g crude water extract. GTE powder was dissolved in distilled water to a concentration of 50 mg/mL and the solution was kept at 4°C.

### Animals

Healthy specific-pathogen-free female Wistar rats (12 weeks of age) were provided by the Laboratory Animal Center of Jilin University and were used for all animal experiments. All rats were raised in polypropylene cages with sterile paddy husk and kept under a controlled environment (humidity 50–60%; ambient temperature 24 ± 1°C; light–dark cycle: 12L:12D). Experimental maintenance rat chow (XieTong Organism, JiangSu, China) based upon the mean weekly food consumption of the sham group was used for feeding OVX rats. The calcium content of our rat chow is about 10–18 g/kg; the phosphorus content is about 6–12 g/kg. All experimental procedures were performed according to the guidelines of the Yunnan Agricultural University Committee for Care and Use of Laboratory Animals and were approved by the Animal Experiments Ethics Committee of Yunnan Agricultural University.

### Group designations and treatment administration

After the rats were allowed to acclimate for 1 week, they were anesthetized with chloral hydrate and underwent resection of the bilateral ovaries (OVX). A further 12 rats (the sham group) were also anesthetized but only underwent resection of a small sample of fat, rather than the bilateral ovaries. All rats were monitored for 15 days before initiating the therapeutic regimen, to allow them to recover from the operation. We randomly divided the OVX rats (*n* = 70) into five groups: model group, XLGB capsule group (240 mg·kg^−1^), low-dose GTE group (low dose, 60 mg kg kg^−1^), medium-dose GTE group (medium dose, 120 mg kg^−1^), and a high-dose GTE group (high dose, 370 mg kg^−1^). Each group contained 14 rats. The animals were continuously administered with their respective treatments via gavage (10 mL/kg) every day for 13 weeks, and an equal volume of distilled water was intragastrically administered to the sham and model groups. XLGB capsules are widely used for the treatment of osteoporosis as a traditional Chinese medicine (3). The dosage of XLGB capsules for rats in our present study was based on the dosage used in clinical trials and calculated by a dose conversion table between human and rats. Furthermore, in the present study, the different dosage levels of GTE were determined and calculated based on previous research (23) and slightly modified to adapt to the current experimental conditions.

We weighed rats weekly during the treatment period. After the treatment period end, we euthanized rats by deep ether anesthesia and obtained blood samples for biochemical analyses. The uterus, left–right femur, and vagina were collected, and the samples for histological analysis were fixed in 10% neutral formaldehyde and then stored at room temperature for subsequent use.

### Analysis of biochemical parameters in blood samples

Blood samples were incubated at room temperature for 2 h and centrifuged at 1,200 g for 10 min at 4°C, then serum was collected and stored at −20°C to await subsequent biochemical analysis. ALP, ACP, BGP, and E_2_ levels were detected in rat serum according to the kit manufacturer’s instructions. In addition, IL-1β and IL-6 levels were detected in the rat plasma.

### Determination of organ coefficients

We completely removed and weighed the left femur and uterus. Then, organ coefficients were calculated as follows: organ coefficient = wet weight of organs/body weight.

### Detection of bone mass and biomechanical testing

The intact left femur of each rat was removed with the muscle and connective tissue was peeled off before analysis. Femoral bone mineral density (BMD) was detected using dual-energy X-ray absorptiometry (LUNAR^®^ Expert #1170, Lunar Prodigy Advance DEXA, GE Healthcare, Madison, WI, USA), in accordance with the manufacturer’s instructions (24). Briefly speaking, the left femur was scanned, and the femoral BMD value was measured automatically. The maximum deflection of the left femur in OVX rats was also evaluated by using the three-point bending flexural test method (24). Therefore, the femur was placed in a biomechanical testing instrument (Changchun Research Institute for Mechanical Science Co., Ltd., Changchun, China) programmed with a stride distance of 20 mm and a loading velocity of 5 mm/s. The data were recorded on a computer, then the maximum deflection was calculated.

### Histological analysis of the uterus and femur

The location of structural analysis of cortical thickness was 1/3 near-end of the right femurs. Fresh femur tissue was collected and fixed in 10% neutral formaldehyde for 72 h, decalcified in ethylenediaminetetraacetic acid (Sigma, St. Louis, Missouri, USA) pH 7.4 for 1 week, and then embedded in paraffin to perform sections following the longitudinal axis. Embedded tissues were cut into 4 μm sections and then were stained with hematoxylin and eosin (H&E) in accordance with a standard technique described previously (25). For analysis of the trabecular bone, consecutive slices (4 μm) were selected as the region of interest beginning 3.5 mm away from the distal femur growth plate. Static structural images of the cortical and trabecular bone were acquired using a medical image analysis system (BI-2000, Taimeng, Chengdu Technology 1 & Market Co., Ltd., Chengdu, China). Cortical bone thickness and trabecular bone area were measured using computer-aided software.

### Cell culture and maintenance

RAW 264.7 murine macrophages (ATCC, Manassas, VA, USA) were used in this study as a cell model. Cells were cultured in Dulbecco’s Modified Eagle’s Medium (DMEM) supplemented with 10% fetal bovine serum (FBS) at 37°C in a humidified atmosphere with 5% CO_2_. DMEM and FBS were purchased from Thermo Fisher Scientific and Biological Industries (Israel BeitHaemek Ltd.), respectively.

### *In vitro* osteoclastogenesis assay

To induce osteoclasts, RAW 264.7 cells (ATCC^®^TIB-71TM, macrophage, Abelson murine leukemia virus transformed, 2 × 10^3^ cells/well) were cultured in the presence of RANKL (50 ng/mL) (26). After 6 days, cells were fixed and then stained for TRAP activity in accordance with the kit manufacturer’s protocol. Cells were defined as mature osteoclasts if light microscopy showed that they were TRAP-positive multinucleated cells with more than five nuclei.

### Quantitative real-time reverse transcription PCR analysis

RAW 264.7 cells (1.2 × 10^5^ cells/well) were inoculated in a 12-well plate and then treated with RANKL (50 ng/mL) in the absence or presence of XLGB (10 μg/mL) or GTE (25, 50, 100 μg/mL) for 48 h. Total RNA was extracted using TransZol Up (TransGen Biotech, Beijing, China) according to the manufacturer’s protocol. Reverse transcription was performed using the PrimeScript RT Reagent Kit with gDNA Eraser (TaKaRa Bio, Otsu, Japan) in accordance with the manufacturer’s protocol. Quantitative real-time reverse transcription PCR (qRT-PCR) was performed using SYBR^®^ Premix Ex Taq™II (TliRNaseH Plus, TaKaRa Bio), and results were determined using a 7900HT Fast Real-Time PCR system (Applied Biosystems, Foster City, CA, USA). Data were calculated using the comparative 2–^ΔΔCT^ method, and all values were normalized to the mRNA level of the endogenous *GAPDH* gene (25). The primer sequences (Generay Biotech, Shanghai, China) are provided in Table 1.

**Table 1:** Primers used in the qRT-PCR study

Genes	Forward (5′–3′)	Reverse (5′–3′)
*GADPH*	AACTTTGGCATTGTGGAAGG	ACACATTGGGGGTAGGAACA
*TRAP*	GCTGGAAACCATGATCACCT	GAGTTGCCACACAGCATCAC
*c-Fos*	CAAGCGGAGACAGATCAACTTG	TTTCCTTCTCTTTCAGCAGATTGG
*cathepsin K*	CTTCCAATACGTGCAGCAGA	TCTTCAGGGCTTTCTCGTTC
*MMP-9*	CGTCGTGATCCCCACTTACT	AACACACAGGGTTTGCCTTC
*NFATc1*	TGGAGAAGCAGAGCACAGAC	GCGGAAAGGTGGTATCTCAA

*Notes*: qRT-PCR, quantitative reverse transcription polymerase chain reaction; TRAP, tartrate-resistant acid phosphatase; NFATc1, activated T cells cytoplasmic 1; MMP, metalloproteinase 9.

### Protein preparation and Western blot analysis

RAW 264.7 cells (4 × 10^5^ cells/well) were inoculated in 60-mm plates and incubated overnight. Then, the cells were treated with RANKL (50 ng/mL) in the absence or presence of XLGB (10 μg/mL) or GTE (25, 50, or 100 μg/mL) for 48 h. Western blot analysis was then performed as previously described (27). In brief, whole cell lysates were prepared from cultured cells using RIPA buffer (Solarbio, Beijing, China) according to the manufacturer’s protocol. Cell lysates were normalized to calculate protein concentration by using the bicinchoninic acid (BCA) method. Then proteins were separated by SDS-PAGE and transferred to polyvinylidene fluoride (PVDF) membranes (EMD Millipore Corporation, Merck Life Sciences, KGaA, Darmstadt, Germany). After washing, blocking, and hatching with the primary antibody, the membrane was hatched with a proper horseradish peroxidase-conjugated secondary antibody, and the resultant bands were detected using a Pro-light HRP Chemiluminescent Kit (Tiangen Biotech, Beijing, China). The representative images were finally acquired using a FluorChem E System (ProteinSimple, Santa Clara, CA).

### Statistical analyses

We presented all data as the mean and standard deviations of the mean (SD). Differences within groups were analyzed statistically using one-way ANOVA and *p* < 0.05 was considered to be statistically significant. All analyses were performed using SPSS 17.0 (Chicago, IL, USA) and GraphPad Prism 5 (GraphPad Software, Inc., La Jolla, CA, USA).

## Results

### GTE influenced the body weight of OVX rats

As shown in Table 2, body weight increased with advancing age. The body weight of the model group increased significantly when compared with the sham group (*p* < 0.01), although the same amount of food was provided to both groups.

**Table 2:** Effect of green tea extract on body weight (g) in OVX rats

Time (week)	Sham	Model	XLGB	Low-dose	Medium-dose	High-dose
0	254.0 ± 17.54 (12)^b^	278.3 ± 22.23 (14)	279.9 ± 19.89 (14)	282.4 ± 21.02 (14)	280.4 ± 28.78 (14)	278.8 ± 24.08 (14)
1	260.3 ± 18.66 (12)^b^	290.0 ± 32.50 (14)	298.0 ± 25.57 (14)	297.4 ± 25.78 (14)	298.9 ± 30.16 (13)	290.1 ± 24.30 (14)
2	263.3 ± 18.87 (12)^c^	295.8 ± 21.21 (13)	304.4 ± 29.58 (14)	304.5 ± 22.50 (13)	300.7 ± 37.19 (13)	282.1 ± 27.16 (14)
3	268.6 ± 17.64 (12)^c^	311.5 ± 23.63 (13)	318.0 ± 27.90 (14)	311.3 ± 26.61 (13)	304.2 ± 47.19 (13)	306.7 ± 33.83 (14)
4	271.8 ± 17.85 (12)^c^	317.5 ± 22.61 (13)	322.2 ± 27.30 (14)	310.8 ± 25.07 (13)	316.5 ± 33.96 (12)	311.4 ± 34.23 (14)
5	275.1 ± 16.08(12)^c^	327.5 ± 17.22 (13)	331.9 ± 29.08 (14)	328.6 ± 24.56 (13)	326.0 ± 33.76 (12)	320.6 ± 33.46 (14)
6	270.3 ± 14.25 (12)^c^	335.9 ± 14.82 (13)	331.0 ± 29.62 (14)	330.6 ± 25.41 (13)	323.9 ± 31.68 (12)	319.4 ± 31.91 (14)
7	278.6 ± 15.59 (12)^c^	343.7 ± 15.16 (13)	336.4 ± 26.45 (14)	340.6 ± 26.86 (13)	328.6 ± 34.67 (12)	328.9 ± 30.44 (14)
8	283.1 ± 24.27 (12)^c^	353.1 ± 16.61 (13)	338.7 ± 31.11 (14)	347.6 ± 37.28 (13)	336.5 ± 36.32 (12)	332.2 ± 32.19 (14)^a^
9	281.0 ± 24.27 (12)^c^	353.6 ± 15.27 (13)	349.9 ± 28.27 (14)	355.2 ± 31.13 (13)	338.5 ± 33.60 (12)	340.5 ± 33.24 (14)
10	284.9 ± 15.5 (12)^c^	351.3 ± 18.25 (13)	351.4 ± 28.41 (14)	352.0 ± 31.73 (11)	344.2 ± 35.40 (12)	342.9 ± 31.00 (14)
11	298.6 ± 16.29 (12)^c^	352.8 ± 32.74 (13)	362.5 ± 30.81 (13)	359.2 ± 32.91 (11)	354.3 ± 35.41 (12)	353.9 ± 32.12 (14)
12	296.5 ± 19.67 (12)^c^	365.9 ± 19.23 (13)	366.1 ± 30.79 (13)	369.2 ± 33.62 (11)	365.2 ± 34.60 (12)	365.4 ± 33.35 (14)
13	303.0 ± 20.85 (12)^c^	367.0 ± 17.68 (13)	366.7 ± 31.14 (13)	365.9 ± 33.67 (11)	367.3 ± 36.38 (12)	364.3 ± 33.16 (13)

*Notes*: All data are presented as mean ± SD (*n* = 12–14).

a *p* < 0.05,

b *p* < 0.01, and

c *p* < 0.001 versus the model group. OVX, ovariectomized; XLGB, Xian-Ling-Gu-Bao.

Body weight did not increase in the XLGB group compared with the model group and there were no significant differences between the GTE groups and the model group during the first 7 weeks of treatment. However, OVX rats treated with high-dose GTE showed a reduction in body weight at Week 8, compared with the model group (*p* < 0.05).

### GTE influenced the serology indicators of PMOP in OVX rats

**Fig. 1:**
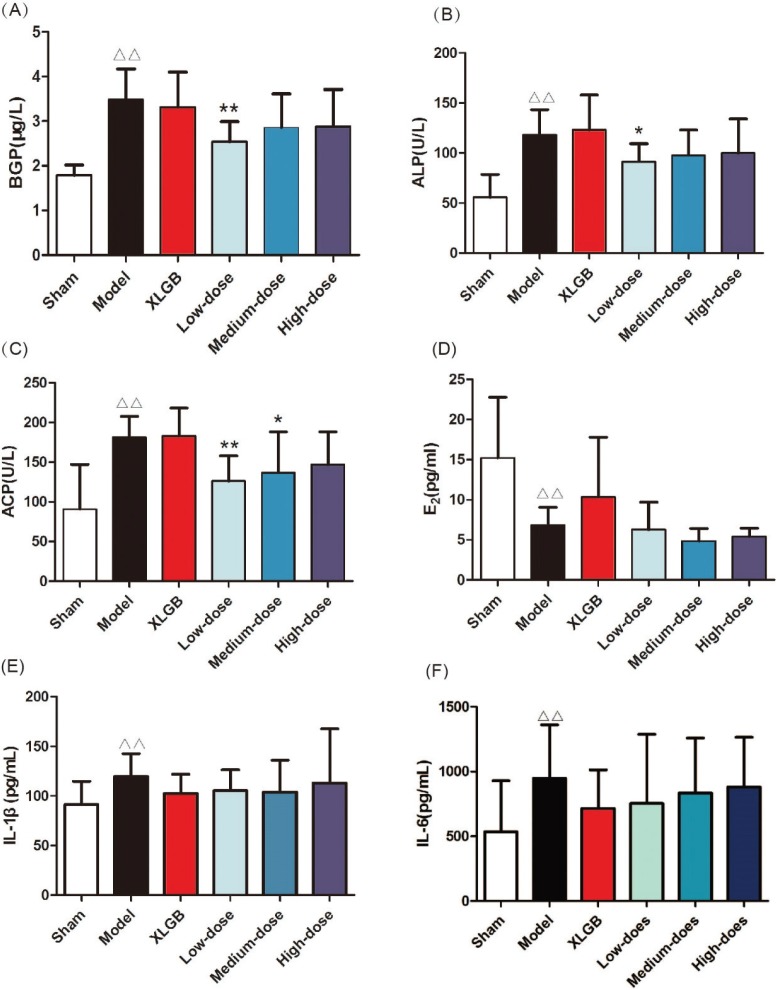
GTE treatment attenuated the serology indicators of postmenopausal osteoporosis (PMOP) in ovariectomized (OVX) rats. (A) Serum bone gla protein (BGP); (B) alkaline phosphatase (ALP) acid; (C) phosphatase (ACP); (D) estrogen (E_2_); (E) interleukin-1β (IL-1β); (F) interleukin-6 (IL-6). All data are presented as mean ± SD (*n* = 10). ^Δ^*p* < 0.05 and ^ΔΔ^*p* < 0.01 *versus* the sham group; and *p <0.05 and **p < 0.01 versus the model group. Sham: underwent resection of a small sample of fat Model: underwent resection of the bilateral ovaries XLGB: Xian-Ling-Gu-Bao capsule group (XLGB, 240 mg kg^−1^) Low-dose: administered GTE (60 mg kg^−1^) Medium-dose: administered GTE (120 mg kg^−1^) High-dose: administered GTE (370 mg kg^−1^)

In order to examine the effect of GTE upon serology indicators of PMOP in OVX rats, serum BGP, ALP, ACP, E_2_, and plasma IL-1β and IL-6 levels were determined using appropriate assay kits (Fig. 1).

Serum BGP, ALP, and ACP were evaluated as biomarkers of bone formation and bone resorption (Fig. 1A–C). In the model group, these parameters were significantly increased compared with those in the sham group (*p* < 0.01). In OVX rats treated with low-dose GTE, the serum BGP and ALP levels were lower than those of the model group (Fig. 1A and B). OVX rats treated with low-dose and medium-dose GTE showed lower serum ACP levels compared with the model group (Fig. 1C).

The level of serum E_2_ in the model group was significantly lower than that in the sham group (*p* < 0.01, Fig. 1D). Compared with the model group, OVX rats treated with XLGB showed moderately increased E_2_ levels. GTE showed no significant influence on serum E_2_ level in OVX rats.

Many cytokines are associated with bone resorption, including IL-1β and IL-6. There were significant differences in IL-1β and IL-6 when comparing levels between the model and sham groups (*p* < 0.05; Fig. 1E and F). The plasma levels of IL-1β and IL-6 in OVX rats treated with XLGB and GTE (at any dose) were lower than in the model group. These results revealed that GTE could improve bone homeostasis in OVX rats.

### Organ coefficients of the femur and uterus in OVX rats

PMOP can lead to a variety of problems with vital organs. For example, we found that following ovariectomy, the uterus of female rats became atrophied and this could also lead to vaginal atrophy. To further investigate the influence of GTE on OVA rats’ organs, we determined the organ coefficients for the femur and uterus of OVX rats.

**Fig. 2:**
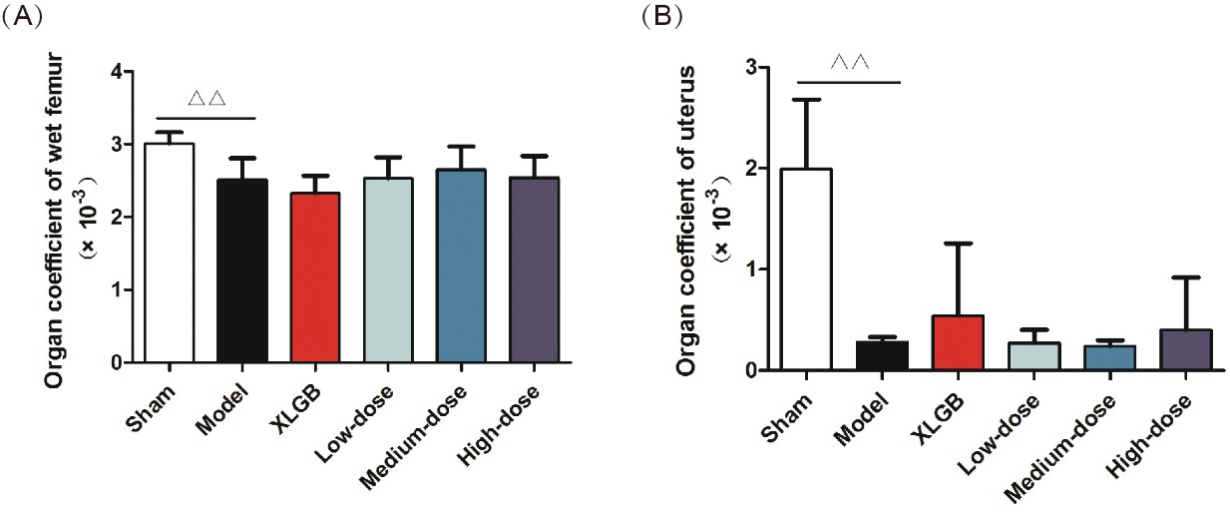
Organ coefficients. (A) Femur and (B) uterus. All data are presented as mean ± SD (*n* = 10). ^Δ^*p* < 0.05 and ^ΔΔ^*p* < 0.01 versus the sham group, and *p < 0.05 and **p < 0.01 versus the model group.

The organ coefficients of the femur and uterus in the model group were all significantly smaller than those of the sham group (*p* < 0.01, Fig. 2A and B). However, compared with the model group, the OVX rats treated with GTE had no obvious effect on either the femur or uterus organ coefficient. These results showed that GTE did not improve the organ coefficients in OVX rats to any extent.

### GTE improved femoral BMD, biomechanical properties, and bone microarchitecture in OVX rats

**Fig. 3:**
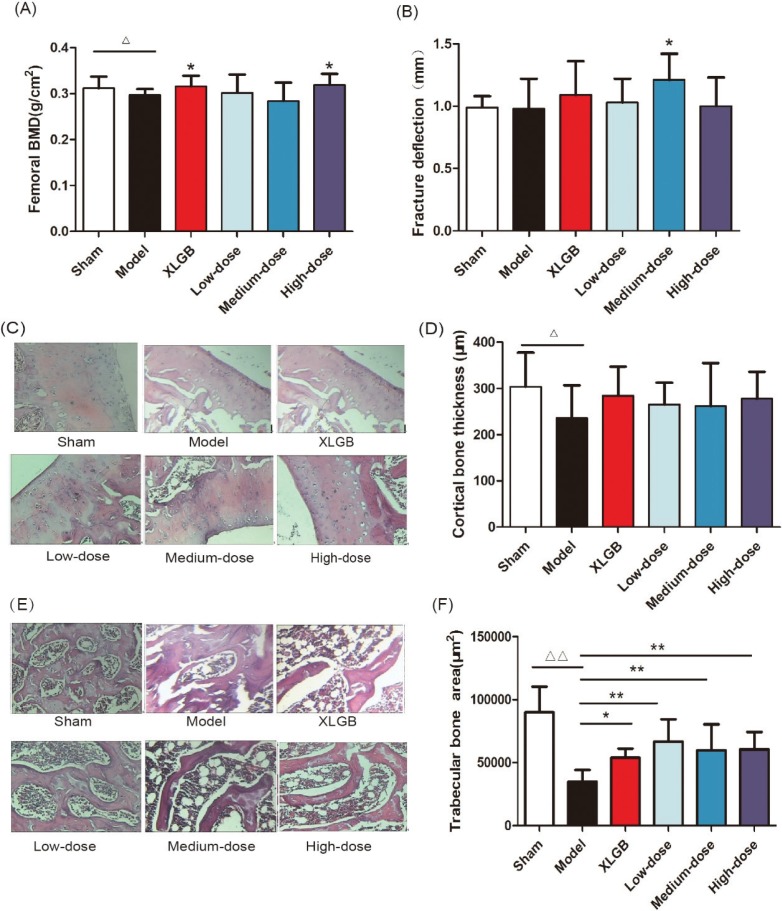
GTE treatment improved femoral bone mineral density (BMD) (A) and fracture deflection (B) in OVX rats. Cortical bone tissue (C) and trabecular bone tissue (E) were stained with H&E; furthermore, the cortical bone thickness (D) and trabecular bone area (F) was calculated. Representative images were acquired using a medical image analysis system, and the original magnification was ×400. All data are presented as the mean ± SD (*n* = 10). ^Δ^*p* < 0.05 and ^ΔΔ^*p* < 0.01 versus the sham group, and *p < 0.05 and **p < 0.01 versus the model group. Scale bar = 40 μm.

We determined the BMD and maximum deflection of the femur in OVX rats to further investigate the protective effects of GTE upon bone (Fig. 3). The femoral BMD of OVX rats decreased significantly to 0.297 ± 0.013 g/cm^2^, compared with 0.312 ± 0.025 g/cm^2^ in the sham rats (*p* < 0.05), and the femoral BMD increased significantly in the high-dose GTE (*p* < 0.05); the positive control (XLGB) showed the same effect (Fig. 3A).

Next, we analyzed the femoral biomechanical properties by determining the maximum deflection. We revealed that the maximum deflection of the medium-dose GTE was significantly increased compared with the model group (*p* < 0.05, Fig. 3B).

**Fig. 4:**
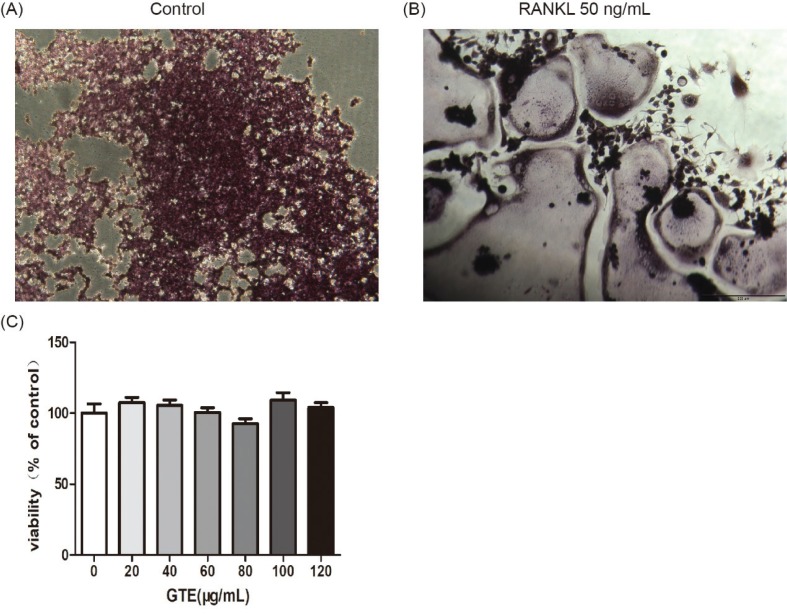
Osteoclast differentiation was induced by receptor activator for nuclear factor-κ B ligand (RANKL) in RAW 264.7 cells (2 × 10^3^ cells/well). After 6 days, cells were fixed and then stained for TRAP activity according to the manufacturer’s protocol (A,B). The effect of GTE treated 48 h on the viability of RAW 264.7 cells as determined by the MTT assay (C). All data are presented as the mean ± SD. *p < 0.05 and **p < 0.01 versus the control group.

**Fig. 5:**
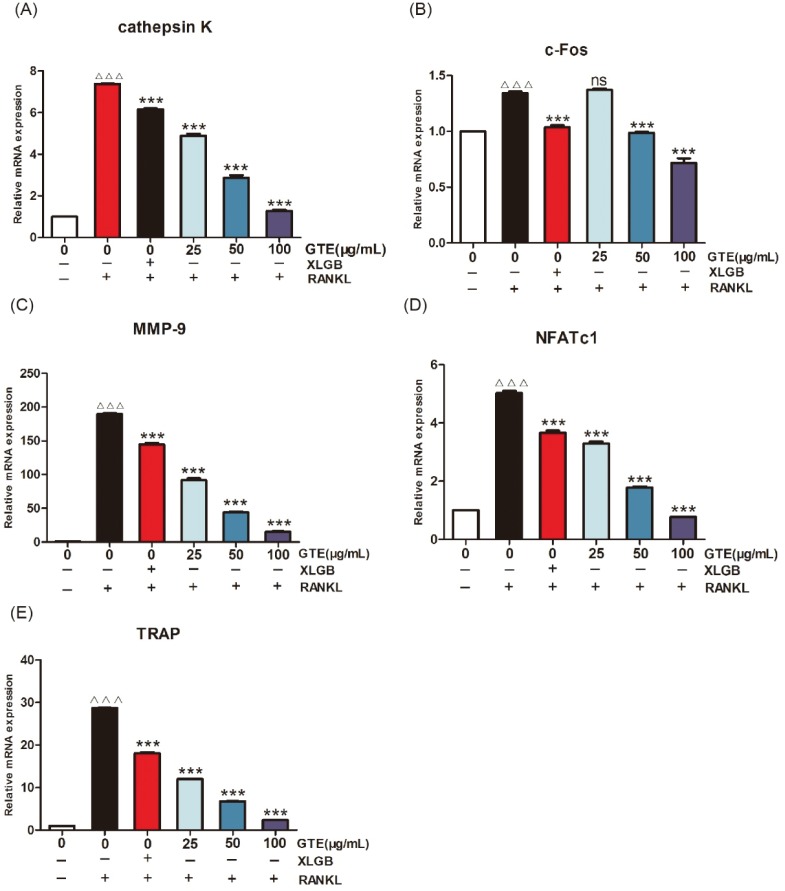
GTE treatment suppressed RANKL-induced osteoclast-specific gene expression. The mRNA expression levels of *cathepsin K* (A), *c-Fos* (B), *matrix metalloproteinase-9* (MMP-9) (C), *NFATc1* (D), and *TRAP* (E). ^ΔΔΔ^*p* < 0.001 compared with control; ***p < 0.001 compared with RANKL treatment only.

**Fig. 6:**
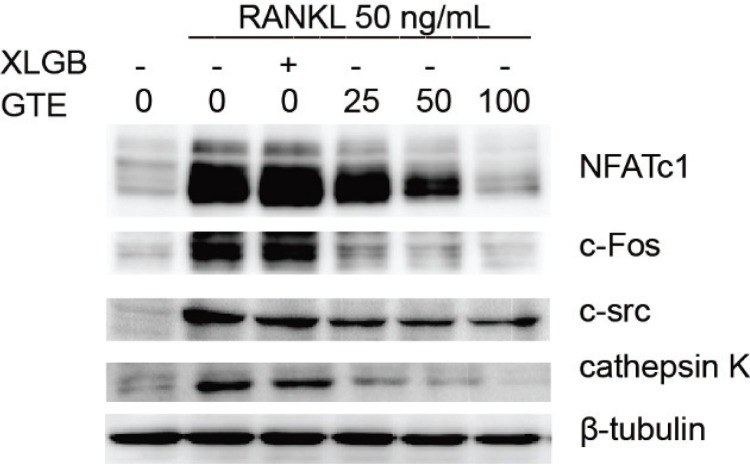
The RANKL-induced osteoclast-specific protein expression of NFATc1, c-Fos, c-Src, and cathepsin K were downregulated by GTE.

These results suggested that treatment with GTE could improve femoral BMD and biomechanical properties in OVX rats. Furthermore, the trabecular bone microarchitectures in the femur were analyzed. As expected, the thickness of the cortical bone and the trabecular bone in the model group was significantly reduced compared to that in the sham group (Fig. 3C and E). Treatment with GTE could increase the thickness of the cortical bone slightly, but these changes had no statistical significance (Fig. 3D). However, treatment with XLGB or GTE led to a significant improvement in the trabecular bone area (*p* < 0.05 or *p* < 0.01; Fig. 3F). In particular, it was evident that the increase in trabecular bone area in the GTE groups occurred in a dose-dependent manner. These results suggested that GTE has a protective effect on bone quality in OVX rats. Furthermore, an osteoclast formation induced by RANKL in RAW 264.7 cells was successfully established (Fig. 4A and 4B). To determine whether GTE had cytotoxicity to RAW 264.7 cells, we further examined the cytotoxicity of GTE in RAW 264.7 cells using the 3-(4,5-Dimethylthiazol-2-yl)-2,5-diphenyltetrazolium bromide (MTT) assay (Fig. 4C). As expected, GTE did not have a cytotoxic effect on osteoclast precursor cells. The qRT-PCR analysis showed that the osteoclastogenesis of *NFATc1*, *c-Fos*, *c-Src*, and *cathepsin K* genes were all inhibited by GTE treatment (Fig. 5). Collectively, Western blotting results indicated that GTE downregulated the expressions of the NFATc1, c-Fos, c-src and cathepsin K protein (Fig. 6).

## Discussion and conclusions

Green tea, a popular well-known beverage worldwide, has captured considerable attention for its scientifically demonstrated beneficial effects on human health (25). Most of these positive and significant effects are attributed to its polyphenolic flavonoids, such as catechins (epicatechin, epigallocatechin, and epicatechin-3-gallate, as well as the major flavonoid (−)-epigallocatechin-3-gallate) (28). The most commonly recognized property of green tea is its potent antioxidant activity, which arises from an ability to scavenge reactive oxygen species (16).

In this study, we used OVX rats to induce a model of PMOP. The primary roles of 17-β estradiol and other estrogens are as regulators of reproductive function (29), although it is now appreciated that these steroid hormones play important activities in other processes that are unrelated to reproduction. Notably, the cessation of ovarian estrogen production that occurs at menopause is associated with an increased risk of osteoporosis, cardiovascular disease, and vasomotor instability (30). Consequently, the OVX model is a highly useful and relevant model for postmenopausal women. Postmenopausal women are commonly linked with weight gain (31). According to previous study, body fat mass is negatively correlated with bone mass when the mechanical loading effect of body weight is statistically removed, suggested that interventions or treatments reducing obesity might increase bone mass and thus protect against osteoporosis (32). In our study, body weight, serum indexes, organ coefficients, and biomechanical parameters were significantly altered compared to the sham group. Collectively, these results showed that the OVX rats represented a useful model of PMOP.

Animal weight was observed throughout the study; high-dose GTE (370 mg kg^−1^) could decrease the body weight at Week 8 compared with model group, which was probably due to the error of measurement or the decrease in food intake. Our data therefore showed that GTE had no obvious effect on weight gain induced by estrogen deficiency. Bone is a complex tissue; its fundamental function is to resist mechanical injury and absorb pressure (33). However, bone strength depends upon the quantity and quality of bone tissue, which is defined by the geometry and shape of the bone, the microarchitecture of the trabecular bone morphology, cortical thickness, and porosity. Bone strength is also characterized by the intrinsic properties of bone tissue such as turnover, mineral composition, and collagen. Furthermore, our present work revealed that GTE treatment could improve the femoral BMD, biomechanical properties, and bone microarchitecture of OVX rats (Fig. 3A–E), implying that GTE could be of significant benefit to patients with PMOP.

Our *in vitro* studies showed that GTE could inhibit RANKL-induced osteoclast-specific gene and protein expression (Figs. 5 and 6). It is well known that osteoclast differentiation is directed by the expression of a range of marker genes, such as *NFATc1*, *TRAP*, *c-Fos*, *c-Src*, *cathepsin K*, and *MMP-9*. Most of these markers are regulated by *NFATc1* (34), a member of the NFAT (nuclear factor of activated T cells) family of transcription factor genes. *NFATc1* is the most strongly induced transcription factor gene following stimulation by RANKL, and it has been heavily implicated in the bone remodeling process in which RANKL-induced osteoclast differentiation plays a central role; if the expression of *c-Fos* increases, then the levels of *NFATc1* are upregulated (35, 36). Furthermore, data derived from our experiments with RAW 264.7 cells showed that *NFATc1* levels could be upregulated by RANKL, while treatment with GTE led to a notable reduction in the expression of RANKL-induced NFATc1. The downregulation of NFATc1 implied that the ability of RAW 264.7 cells to turn into osteoclasts had been suppressed by GTE treatment (Fig. 4B). Cathepsin K is a cysteine proteinase expressed predominantly by osteoclasts; this enzyme cleaves key bone matrix proteins and is believed to play an important role in degrading the organic phase of bone during bone resorption (37). Studies have shown that the overexpression of cathepsin K can perturb bone metabolism and thus cause bone loss. GTE also could reduce the expression of cathepsin K induced by RANKL, meaning that GTE could reduce bone loss by suppressing the expression of cathepsin K (Fig. 4B). C-Fos and MMP-9 are other examples of transcription factors involved in the formation of osteoclasts induced by RANKL. According to our current data, the expression of c-Fos and MMP-9 was also reduced in response to GTE treatment. In general, our study showed that GTE treatment suppressed the expression of osteoclast-specific genes and proteins.

In summary, our data demonstrated that GTE could improve a series of health problems induced by menopause, such as weight gain and organ pathology. Serum tests also showed that GTE could relieve bone loss. We also demonstrated that GTE could improve osteoporosis by suppressing osteoclast-specific gene and protein expression.

It has been reported that green tea aqueous extract consists of many chemical components, including green tea polyphenols (38), caffeine, aromatic oils, pigments and amino acids. Because of the extraordinary complexity of its components, the precise bioactive ingredient(s) responsible for the effect upon postmenopausal osteoporosis remains unidentified. Therefore, further research is urgently required in order to investigate the bioactive components responsible for the anti-osteoporotic effect of green tea. Our current results provided a theoretical basis for the subsequent exploration of the specific chemical constituents contained in green tea extract, which could act up on osteoporosis.
